# Supplementary figures and images for: Individual differences in human voice pitch are preserved from speech to screams, roars and pain cries
Source: R Soc Open Sci. 2020 Feb 26;7(2):191642. doi: 10.1098/rsos.191642 (PMC7062086; doi:10.1098/rsos.191642)

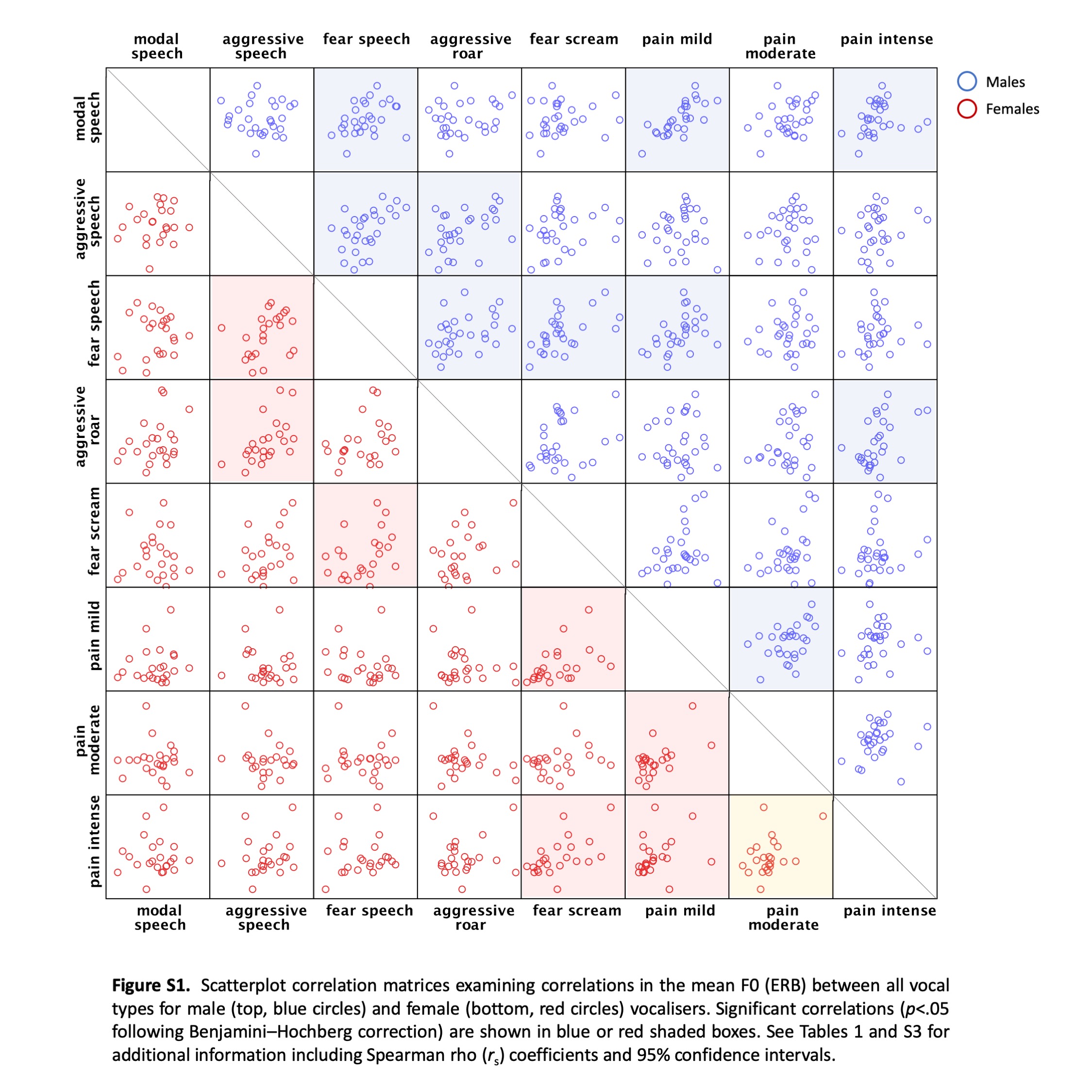

Supplement: Figure S1 [file rsos191642supp3.jpg]
